# Editorial: IL-27 in health and disease

**DOI:** 10.3389/fimmu.2023.1191228

**Published:** 2023-03-31

**Authors:** Tomozumi Imamichi, Xue-Feng Bai, Cory Robinson, Katrina Gee

**Affiliations:** ^1^ Applied Development and Research Directorate, Frederick National Laboratory for Cancer, Frederick, MD, United States; ^2^ Department of Pathology, College of Medicine, The Ohio State University, Columbus, OH, United States; ^3^ Department of Microbiology, Immunology, and Cell Biology, West Virginia University, Morgantown, WV, United States; ^4^ Department of Biomedical and Molecular Sciences, Queen’s University, Kingston, ON, Canada

**Keywords:** IL-27, HIV, inflammation, *Mycobacterium tuberculosis*, cancer

IL-27 is an immunoregulatory cytokine belonging to the IL-12 and IL-6 cytokine families. Identified in 2002, this cytokine is heterodimeric, composed of EBI3 and p28 subunits, and interacts with a heterodimeric receptor composed of WSX-1 and gp130. Produced mainly by antigen-presenting cells in response to stimulation of pattern recognition receptors, early roles for this cytokine were identified to be in the promotion of naïve CD4 T cell proliferation and Th1 differentiation. However, a wide spectrum of different functions for this cytokine quickly became apparent and have since been identified to range from promoting or curbing inflammatory diseases, cancers, and viral infections. Due to the various roles of this cytokine in modulating inflammatory responses, consideration has been given towards the development of therapeutics that either promote its expression or antagonize its function.

The goal of this Research Topic was to further clarify the role of IL-27, including WSX-1/gp130 expression and signaling as well as responses to IL-30 (IL-27p28) across a broad range of inflammatory conditions and diseases. IL-27 expression has been associated with a diverse set of outcomes that appear to be dependent upon the disease or infection, cell activation status, and cell type examined.

IL-27 functions as an anti-viral cytokine in the setting of a variety of different viruses. In this article collection, Li et al. describe how IL-27 impacts cross-talk between T cells and osteoclasts during HIV infection. Using a co-culture of autologous osteoclasts and ex-vivo TCR-stimulated T cells obtained from people with HIV (PWH), they found that IL-27 inhibited IL-17 production in cells cultured from PWH compared to healthy controls. Similarly, IL-27 was found to inhibit RANKL in TCR-stimulated CD4 T cells in co-culture with osteoclasts. However, IL-27 did not affect the expression of other inflammatory cytokines induced by TCR stimulation, namely IFNγ and TNFα, suggesting that IL-27 could function to inhibit TH17 function while promoting Th1 cell function. In the setting of bone remodeling and chronic inflammation during HIV infection, this study indicates that IL-27 could play a role in the modulation of the interactions between osteoclasts and T cells, and this represents an important area for future consideration.

Investigation into the roles of IL-27 in the setting of cancer has led to the finding that this cytokine has differential functions that are context-dependent. Having both pro- and anti-cancer activities as well as direct effects on cancer cells and immune cells, IL-27 expression and function is important to understand. In this article collection Dong et al. provide critical RNA seq data describing the role of *IL27* in the setting of melanoma, where they linked *IL27* expression with survival in patients with melanoma. They also found that *IL27* was associated with the upregulation of several key signaling pathways involved in immune activation as well as cell death, as well as with potential enhancement of immunotherapy.

IL-27 is known to also play divergent roles in the control of inflammation. Watanabe et al. provide a thorough review of the novel role of the IL-27 subunit, EBI3 during inflammatory conditions. Interestingly, EBI3 has recently been attributed chaperone-like functions that has implications in the formation of heterodimeric cytokines, such as IL-27 and related cytokines IL-35 and IL-39, which each incorporate the EBI3 subunit. The inflammatory role of IL-27 in the setting of tuberculosis is further discussed in the review article by Ritter et al. It is interesting to note that a dichotomous role for IL-27 arises yet again, where IL-27 can be both beneficial and detrimental during *Mycobacterium tuberculosis* (Mtb) infection. This review of the literature highlighted that in the absence of IL-27 signaling, mice develop more matured and effective granulomas prior to chronic infection with T cells that exhibit a multifunctional cytokine signature. This was consistent with higher frequencies of IL-17A-expressing Th17 cells. However, during late chronic infection, exaggerated inflammation compromises granuloma organization. This review further presents the idea of IL-27 as a temporal host-directed target not only for therapeutic intervention of TB disease, but potentially prevention through vaccination as well. The dichotomous nature of IL-27 is further highlighted in the review article by Han et al., who detail how IL-27 influences the immune response to Mtb in protective and detrimental manners.

The manuscripts in this Research Topic encompass the role of IL-27 across a broad range of research areas including viral infection, cancer, inflammation, and bacterial disease. The editors of this Research Topic sincerely thank all reviewers and authors for contributing to this topic.

## Author contributions

KG drafted the editorial. X-FB, CR and TI provided input and editing. All authors contributed to the article and approved the submitted version.

